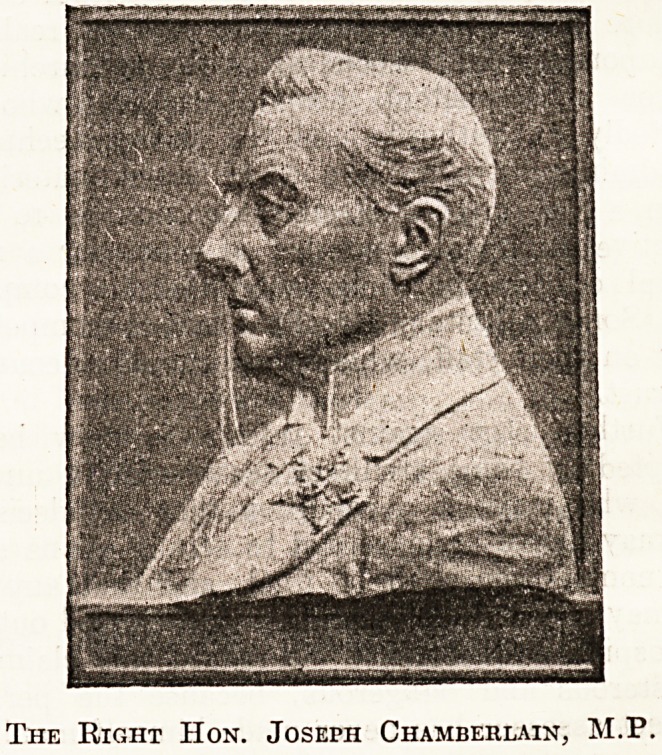# The Chamberlain Memorial: Mr. Harcourt at the Albert Dock Hospital

**Published:** 1914-06-27

**Authors:** 


### THE CHAMBERLAIN MEMORIAL.

### Mr. Harcourt at the Albert Dock Hospital.

A CEREMONY of much interest took place on Tuesday last at the Albert Dock Hospital. This institution, a branch of the Seamen's Hospital Society, to which the London School of Tropical Medicine is attached, was *en fête* on the occasion of the unveiling of bronze portrait reliefs of Mr. Joseph and Mr. Austen Chamberlain. The ceremony was performed by Mr. Lewis Harcourt, Secretary of State for the Colonies.

This memorial has been erected by the Committee of Management of the Seamen's Hospital Society in recognition of the services of both these statesmen to tropical medicine. It will be in the recollection of our readers that recently Mr. Austen Chamberlain raised a fund of £73,000, in addition to large funds raised by his father, for the founding of the school and for its enlargement.

THE RIGHT HON. AUSTEN CHAMBERLAIN, M.P.

The memorial is believed to be one of the most handsome that can be found in any great public institution. It consists, as our illustrations show, of two bronze portrait reliefs, about 11 inches by 8 inches each, which are characteristic representations of Mr. Joseph Chamberlain and his son. These are mounted on grey-figured marble, with the following inscription beneath :—

"CHAMBERLAIN WARD."
Named in commemoration of the invaluable services
rendered to Tropical Medicine by
The Rt. Hon. Joseph Chamberlain, M.P.,
and
The Rt. Hon. Austen Chamberlain, M.P
1913.

The reliefs have been designed and executed by Mr. F. W. Doyle Jones.

About 200 guests assembled and were received by the chairman of the committee of management, Mr. Percival A. Nairne. The wards were on view as well as the laboratories. In the latter there were shown under the microscope various parasites of tropical disease, including malaria, sleeping sickness, etc.

Tea was served in the large dining hall, which is one of the apartments lately added to the school. There were present, besides Mr. Lewis Harcourt and Mr. Austen Chamberlain :—

THE RIGHT HON. JOSEPH CHAMBERLAIN, M.P.

Mrs. Harcourt, Mrs. Joseph Chamberlain and Mrs. Endicott, Mrs. Austen Chamberlain, Sir John Anderson, G.C.M.G., Permanent Under Secretary of State for the Colonies, and Mrs. Anderson, Sir David Bruce, C.B., Sir Wm. and Lady Bennett, Sir M. M. Bhownaggree, K.C.I.E., Sir Henry Burdett, K.C.B., K.C.V.O., Sir T. Fowell Buxton, G.C.M.G., Sir George and Lady Dashwood, Sir George Denton, K.C.M.G., Sir Thos. and Lady Holderness, Sir Frederick and Lady Lugard, Mr. A. C. Lampard, Mr. Eric Müller, Dr. Roland Brinton, Mr. H. J. Read, C.B., C.M.G., Dr. and Mrs. Sandwith, Mr. and Mrs. James Cantlie, Col. and Mrs. Alcock, Professor R. Tanner Hewlett and Miss Hewlett, Dr. and Mrs. G. C. Low, Dr. C. W. Daniels, Col. and Mrs. J. J. Pratt, and Dr. H. B. Newham.

## Figures and Tables

**Figure f1:**
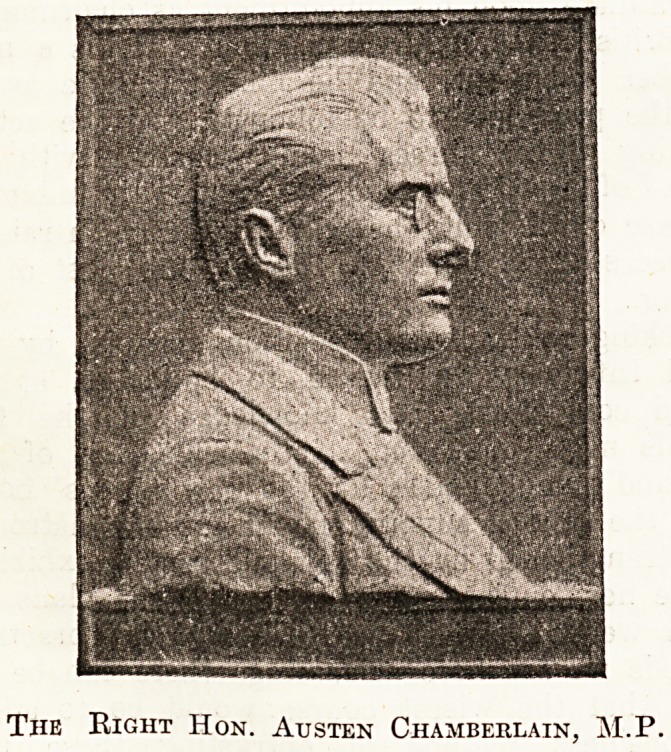


**Figure f2:**